# Seasonal Oscillation of Human Infection with Influenza A/H5N1 in Egypt and Indonesia

**DOI:** 10.1371/journal.pone.0024042

**Published:** 2011-09-01

**Authors:** Eleanor J. Murray, Stephen S. Morse

**Affiliations:** Department of Epidemiology, Mailman School of Public Health, Columbia University, New York, New York, United States of America; University of Zaragoza, Spain

## Abstract

As of June 22, 2011, influenza A/H5N1 has caused a reported 329 deaths and 562 cases in humans, typically attributed to contact with infected poultry. Influenza H5N1 has been described as seasonal. Although several studies have evaluated environmental risk factors for H5N1 in poultry, none have considered seasonality of H5N1 in humans. In addition, temperature and humidity are suspected to drive influenza in temperate regions, but drivers in the tropics are unknown, for H5N1 as well as other influenza viruses. An analysis was conducted to determine whether human H5N1 cases occur seasonally in association with changes in temperature, precipitation and humidity. Data analyzed were H5N1 human cases in Indonesia (n = 135) and Egypt (n = 50), from January 1, 2005 (Indonesia) or 2006 (Egypt) through May 1, 2008 obtained from WHO case reports, and average daily weather conditions obtained from NOAA's National Climatic Data Center. Fourier time series analysis was used to determine seasonality of cases and associations between weather conditions and human H5N1 incidence. Human H5N1 cases in Indonesia occurred with a period of 1.67 years/cycle (p<0.05) and in Egypt, a period of 1.18 years/cycle (p≅0.10). Human H5N1 incidence in Egypt, but not Indonesia, was strongly associated with meteorological variables (κ^2^≥0.94) and peaked in Egypt when precipitation was low, and temperature, absolute humidity and relative humidity were moderate compared to the average daily conditions in Egypt. Weather conditions coinciding with peak human H5N1 incidence in Egypt suggest that human infection may be occurring primarily via droplet transmission from close contact with infected poultry.

## Introduction

Influenza is among the best known and studied of human diseases, yet it remains a major cause of morbidity and mortality [1]. In the United States alone influenza is responsible for between 36,000 [2] and 50,000 [3] deaths on average each year, and millions of cases of disease [3], with 10–20% of the entire population of the US infected [4] in a typical year. A severe pandemic of a novel influenza strain, such as the 1918 Spanish Flu, could result in as many as 1.9 million deaths in the US [5]. Worldwide, 2 billion people have been projected to fall ill during a severe pandemic, a billion of whom are projected to need medical care, with 42 million projected fatalities [5]. The recent appearance of H5N1 influenza in humans has raised concerns that it may have comparable pandemic potential. Although H5N1 primarily infects humans directly from infected birds and sustained human-to-human transmission has not occurred, there have been reports suggesting limited human-to-human transmission, for example between family members [6].

To date, considerable effort has been expended monitoring the genetics of H5N1 in order to identify viral variants capable of pandemic infection; less research has addressed the epidemiology of human infection with H5N1. In particular, despite recent suggestions that H5N1 incidence in humans is seasonal [7], [8], [9], [10], with more cases occurring in cooler months, evidence for these claims has not been critically evaluated, nor has the role of potential environmental drivers of infection seasonality in humans been considered.

The role of static environmental factors in H5N1 outbreaks in poultry, however, have been investigated, including proximity to bodies of water [11], [12], [13], [14] and major highways [11], [13], elevation [11], [13], [14], [15], and farm conditions, such as biosecurity [12], [16] and poultry density [11], [12], [16]. Of these, the environmental factors associated with H5N1 outbreaks in poultry are all indicators of decreased rainfall or the presence standing water, including rivers or streams [11], [13], [14], [17]. In particular, Fang et al. [11] found that each 100 mm increase in total annual precipitation was associated with a 0.9-fold reduction in odds of H5N1 poultry outbreaks (95% CI: 0.87–0.95) in China. While the evidence suggests a role for rainfall in H5N1 incidence in poultry, no studies have considered the impact of seasonal variation in this or other weather conditions on H5N1 incidence in either poultry or humans.

### Influenza seasonality

Human influenza incidence peaks in the Northern and Southern Hemispheres during their respective winters [18], yet, despite recognition of this phenomenon for at least a hundred years [19], the mechanisms driving influenza seasonality are not well understood [20], [21], [22]. Several competing hypotheses have been proffered, including biological, sociological and environmental explanations, but none have been definitively established [22].

The pattern of influenza seasonality in humans appears different in tropical and subtropical areas, with high year-round circulation and semi-annual peaks in incidence [23], [24], [25]. However, in the tropics, understanding the seasonal pattern of influenza in humans is further hampered by a lack of routinely collected incidence data [25]. H5N1 incidence data in humans is collected by active surveillance, and data on this viral subtype therefore is likely more complete than for seasonal influenza. Finally, H5N1 infection in humans rarely occurs via person-to-person transmission, with most human cases occurring due to exposure to infected poultry, reducing the impact of several sociological explanations on seasonality of this strain. Therefore analysis of H5N1 infection in humans may shed light on influenza seasonality and transmission in tropical and sub-tropical regions, especially the role of physical or environmental factors, as well as on the patterns of transmission specific to H5N1 infection.

This study analyzed the role of climate on incidence of human H5N1 in Indonesia and Egypt, and investigated the hypothesis that human H5N1 cases occur seasonally, associated with decreases in temperature, humidity and precipitation.

## Results

### Seasonal oscillation of human H5N1 incidence

**Figure 1:**
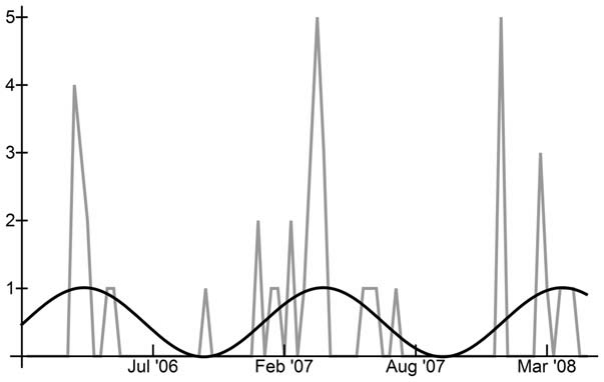
Human H5N1 in all Egypt, Jan 1st, 2006–May 1st, 2008, and comparison with Fourier analysis curves. Black line: sinusoidal curve calculated from Fourier analysis maximum periodogram ordinate based on binary H5N1 incidence data; Grey line: total human H5N1 cases per ten-day interval.

**Figure 2:**
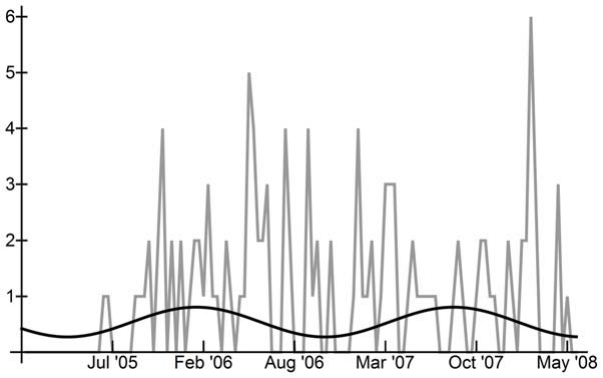
Human H5N1 in all Indonesia, Jan 1st, 2005–May 1st, 2008, and comparison with Fourier analysis curves. Black line: H5N1 incidence sinusoidal curve calculated from Fourier analysis maximum periodogram ordinate based on binary H5N1 incidence data; Grey line: total human H5N1 cases per ten-day interval.

Figures 1 and 2 show the total number of reported cases of human H5N1 occurring in each ten-day interval in Egypt and Indonesia, respectively. In Egypt, human H5N1 cases peaked in late winter and early spring, with more cases occurring in the first 10 intervals of each year, suggesting a seasonal distribution of approximately 12 months (Figure 1). In Indonesia, seasonality of ten-day incidence between January 1^st^, 2005 and May 1^st^, 2008 was less clear (Figure 2), as cases occurred almost constantly in the 18 months following the initial human case.

Fourier analysis of the time series of binary human H5N1 incidence confirmed that human incidence oscillated predictably in Indonesia and in Egypt (Table 1; Figures 1 and 2; Supplementary Figures S1 and S2). In Egypt, the overall pattern of incidence was statistically different from ‘white noise’ (Bartlett's Komolgorov-Smirnov test: p = 0.0199), but the main frequency (maximum periodogram ordinate) was only marginally significant (Fisher's kappa: p≅0.10) (Table 1). The main frequency of oscillation of human cases in Egypt indicated a period of 1.18 years per cycle or approximately 14 months between incidence peaks. In Indonesia, the pattern of cases was found to be significantly different from a ‘white noise’ pattern (Bartlett's Komolgorov-Smirnov test: p<0.05) and the main frequency identified by Fourier analysis was statistically significant (Fisher's kappa: p<0.05) (Table 1). Human cases in Indonesia oscillated with a period of 1.67 years per cycle or approximately 18 months between peak incidence levels. For background on significance tests, please see the *Methods* section.

**Table 1:** Parameters from Fourier analysis of single time series.

Variable	Country	Maximum Periodogram Ordinate	Fisher's Kappa†	Fisher's Kappap-value	Bartlett's Test†	Bartlett's p-value	Frequency(radians)	Period(yr/cycle)†
Human H5N1 Cases	Egypt	2.33	5.66	≥0.10	**0.234**	0.0199	0.146	1.178
	Indonesia	4.02	**7.93**	0.01–0.05	**0.276**	0.0002	0.103	**1.671**
Mean Daily Precipitation	Egypt	0.685	**7.23**	0.01–0.05	**0.261**	0.0065	0.146	**1.178**
	Indonesia	518.212	**24.66**	<0.01	**0.479**	<0.0001	0.155	**1.114**
Mean Daily Temperature	Egypt	1909.710	**26.25**	<0.01	**0.834**	<0.0001	0.146	**1.178**
	Indonesia	9.531	**12.85**	<0.01	**0.526**	<0.0001	0.361	**0.477**
Mean Daily Relative Humidity	Egypt	1124.201	**14.95**	<0.01	**0.613**	<0.0001	0.146	**1.178**
	Indonesia	1056.441	**30.79**	<0.01	**0.688**	<0.0001	0.155	**1.114**
Mean Daily Absolute Humidity	Egypt	1116.712	**27.50**	<0.01	**0.878**	<0.0001	0.146	**1.178**
	Indonesia	73.430	**16.86**	<0.01	**0.691**	<0.0001	0.155	**1.114**

† Values in bold are associated with statistical significance at p≤0.05.

### Meteorological drivers of human H5N1 incidence

**Figure 3:**
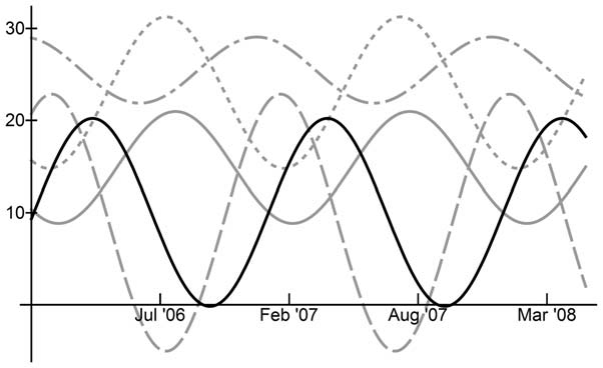
Comparison of human H5N1 incidence seasonality and meteorological seasonality in Egypt using sinusoidal curves calculated from Fourier analysis maximum periodogram ordinates. Solid black line: H5N1 incidence, based on binary H5N1 incidence data; Dotted grey line: Temperature (°C); Dashed grey line: Precipitation (mm); Dash-dot grey line: Relative humidity (%); Solid grey line – Absolute humidity (% vapor pressure). Curves for H5N1 incidence, precipitation and relative humidity have been scaled to fit y-axis, by factors of 40, 80 and 0.5, respectively.

**Figure 4:**
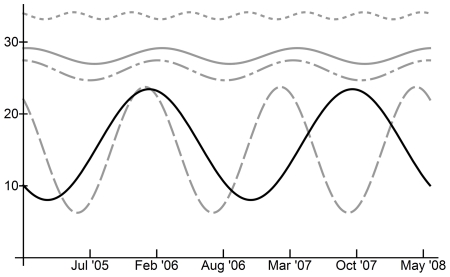
Comparison of human H5N1 incidence seasonality and meteorological seasonality in Indonesia using sinusoidal curves calculated from Fourier analysis maximum periodogram ordinates. Solid black line: H5N1 incidence, based on binary H5N1 incidence data; Dotted grey line: Temperature (°C); Dashed grey line: Precipitation (mm); Dash-dot grey line: Relative humidity (%); Solid grey line – Absolute humidity (% vapor pressure). Curves for H5N1 incidence, precipitation, temperature and relative humidity have been scaled to fit y-axis, by factors of 30, 3, 0.80 and 0.33, respectively.

In Egypt, all meteorological variables (temperature, precipitation, relative humidity and absolute humidity, measured as vapor pressure) exhibited statistically significant seasonal patterns with period length 1.18 years/cycle or 14 months per cycle (Table 1), the same as that observed for human incidence. Using the squared coherency measure, which evaluates the degree of linear relation between variables in cross-spectra, we found strong linear relationships between the time series of human H5N1 incidence and the time series of each of the four meteorological variables (squared coherence range: 0.95 to 0.96; Supplementary Figure S3) (Table 2). Graphical comparison of sinusoidal curves calculated from the main frequencies as determined by Fourier analysis (note that these equations are scaled to fit the same ordinate axis; see Table S1 for un-scaled curve equations) clearly demonstrated a relationship between all four meteorological variables and human H5N1 incidence (Figure 3). Across Egypt, daily ranges for meteorological variables were 12–32°C for temperature, 38–65% for relative humidity, 8–23% for absolute humidity (vapor pressure) and 0–1.1 mm for precipitation (Table 3). Peak H5N1 human incidence in Egypt coincided with the following meteorological conditions: temperature 20°C, precipitation 0.2 mm, relative humidity 49%, and absolute humidity (vapor pressure) 11% (Table 3). However, the effect of changes in meteorological conditions on reported human H5N1 incidence is anticipated to take 6–8 weeks to occur, given the time required for transmission and incubation before cases are reported. At 7 weeks prior to peak human H5N1 incidence, the meteorological conditions were: temperature 15°C, precipitation 0.3 mm, relative humidity 55%, and absolute humidity (vapor pressure) 9% (Table 3). In Indonesia daily temperatures during the study period ranged from 25 to 29°C; relative humidity (RH) ranged from 65 to 85%; absolute humidity (vapor pressure), ranged from 23 to 31%; and precipitation from 0.2 to 14.7 mm (Table 3). However, seasonality of meteorological conditions was not useful for predicting H5N1 incidence in Indonesia (Figure 4). The relationship between H5N1 incidence and meteorological variables in Indonesia could not be compared directly by computing the squared coherency at the main frequency since the time series oscillated at different frequencies (Table 1). Instead, the squared coherencies were computed over all frequencies for these time series; for the range of frequencies observed in the incidence and meteorological data, the squared coherencies indicated at best a weak linear relationship (Figure S1; squared coherency range: 0.2–0.6).

**Table 2:** Squared coherency (κ^2^) values evaluated at the main frequency for cross-spectra between human H5N1 cases and weather variables, in Egypt.

Data series	Squared Coherency (κ_x,y_^2^) with Human H5N1 Incidence
Precipitation	0.949
Temperature	0.953
Relative Humidity	0.963
Absolute Humidity	0.949

**Table 3:** Meteorological conditions in Indonesia and Egypt during the study period (range), and coincident with, and at 7 weeks prior to, peak H5N1 incidence in humans.

Country	Data series	Minimum Value During Study Period	Maximum Value During Study Period	Value at Peak H5N1 Incidence	Value 7 Weeks Prior to Peak H5N1 Incidence*
Indonesia	Precipitation	0.2 mm	14.7 mm	n/a	n/a
	Temperature	25.2°C	28.2°C	n/a	n/a
	Relative Humidity	65.7%	84.7%	n/a	n/a
	Absolute Humidity	23.8%	30.2%	n/a	n/a
Egypt	Precipitation	0.0 mm	1.1 mm	0.19 mm	0.28 mm
	Temperature	12.2°C	32.1°C	19.84°C	15.18°C
	Relative Humidity	37.7%	64.8%	48.78%	54.76%
	Absolute Humidity	7.6%	23.4%	11.22%	8.85%

*Calculated based on five 10-day intervals, given an expected delay between meteorological change and human H5N1 reporting of 6–8 weeks.

## Discussion

### Seasonal oscillation of human H5N1 incidence

Although the small number of cases and short time span available may have reduced the significance level of results, H5N1 in humans appeared to oscillate seasonally in both Egypt and Indonesia. In Indonesia, H5N1 incidence peaked every 18 months, but was not obviously related to climate conditions, which oscillated over shorter periods. This apparent lack of a relationship between meteorological conditions and human H5N1 incidence in Indonesia is an important finding, but one that may be explained by local variation in microclimate or in human or poultry susceptibility to H5N1, or by reduced seasonal variability in meteorological conditions. Further, changes in meteorological conditions may alter contact between humans and poultry differently, in different regions. Indeed, meteorological conditions were more varied throughout Indonesia than Egypt, and the assumption that country-wide average conditions were sufficient may not have been appropriate for Indonesia. Further research using more fine-grained meteorological and case data may resolve this issue.

In Egypt, H5N1 incidence oscillated on a 14 month cycle, the same period as the meteorological variables. Peak human H5N1 incidence in Egypt coincided with specific weather conditions for the period 2006–2008: low precipitation and relatively moderate absolute humidity, temperature and relative humidity levels compared to the average daily conditions for Egypt. These weather conditions varied minimally across Egypt during the study period, with the most significant geographic differences seen in levels of relative humidity. Although the main period of oscillation for human incidence in Egypt was not statistically significant at the 5% level, this may be attributable to the small number of cases which occurred during the study period. Importantly, the overall Fourier analysis spectrum was determined to be significantly different from white noise, and the cross-spectral analysis demonstrated the high level of association between human incidence and meteorological variables.

Little is known about the seasonality of influenza viruses in tropical and sub-tropical countries, where temperature fluctuations are generally less extreme than in temperate regions. Longitudinal studies in Nicaragua [24] and Hong Kong [23] have demonstrated the importance of influenza year-round as a cause of respiratory infections in children, but there is evidence that seasonal peaks in incidence of human influenza do occur, and appear to vary in number and timing between countries and from year to year. Several studies have documented tropical and sub-tropical influenza occurring in semiannual peaks in either the spring and fall, between the influenza seasons in Northern and Southern Hemispheres [25], [26], [27], [28], [29], or the summer and winter [30], [31], [32]. However, other evidence points to the occurrence of only a single peak in seasonal influenza incidence per year [23], [33], [34], or of variation in the number and timing of peaks annually [24], [35]. This variation in timing and number of peaks could be the result of seasonal influenza oscillating with a period of greater than 12 months. The observed periodicity of human H5N1 in Egypt (14 months) and Indonesia (18 months) is consistent with such a model of influenza incidence in tropical and sub-tropical regions.

### Meteorological drivers of human H5N1 incidence

The observed correlation between human H5N1 incidence and meteorological variables in Egypt is surprising given known levels of seasonal influenza virus survival and transmissibility over a range of temperature and humidity levels. For example, aerosol transmissibility of seasonal influenza is reported to be highest at low temperature (optimum: 8°C) [36] and low humidity, both relative (optimum: 25%) [36] and absolute (optimum: <12%) [37], although transmission is moderate at 65% RH and 20°C compared to higher temperature and humidity levels [20].

In Indonesia, weather conditions routinely exceeded optimal conditions for aerosolized virus transmission, which may partially explain the lack of a detected relationship between weather and human H5N1 incidence in Indonesia.

In Egypt, meteorological conditions did approach the favorable ranges for virus survival and aerosol transmission during the study period [37]. However, time periods when conditions in Egypt were expected to be most favorable for aerosolized transmission, based on temperature and relative humidity, did not coincide with peak human H5N1 incidence. Based on survival curves of aerosolized influenza [36], exposure to the weather conditions at and prior to the human H5N1 incidence peak in Egypt (20°C and 49% RH at peak, and 15°C and 55% RH 7 weeks prior) should result in approximately 30–40% of influenza released into the air remaining viable after one hour, while under optimal conditions 78% of airborne influenza would remain viable after one hour [36].

The current analyses support the suggestion that absolute humidity may play a central role in influenza transmission in Egypt, since peak H5N1 incidence coincides with an optimal absolute humidity value of 11%, and absolute humidity values 7 weeks prior to human incidence peak were an even more favorable 9% (optimum: <12%). A recent study of influenza transmission in guinea pigs also found that low absolute humidity was associated with longer influenza virus survival and higher transmissibility of viral particles, independent of the effect of temperature [37]. In addition, there is some evidence that stable temperatures, as occur in Indonesia, are associated with higher influenza mortality [19]; this effect may interact with variations in other meteorological factors to create the observed seasonal patterns of influenza incidence.

Given that temperature and relative humidity appear to be generally unfavorable for aerosolized transmission of influenza during most of the year in Egypt and Indonesia, especially during and immediately preceding times of peak H5N1 incidence, it appears likely that H5N1 transmission is occurring through large droplet or fomite transmission rather than via aerosolized fine particles. A recent paper by Lowen et al [38] demonstrated that contact transmission of seasonal influenza virus, such as through droplets or fomites, can be highly successful at 30°C regardless of relative humidity levels even though fine particle aerosol transmission does not occur readily at these conditions [38]. In addition, although survival of seasonal influenza on laboratory surfaces is typically low at climate conditions observed in Indonesia and Egypt [39], [40], researchers have previously isolated infectious H5N1 virus from the environment, including from standing water and bird feces [41], [42], supporting a role for fomite transmission. However, the relative roles of large droplets, fine particle aerosols, and fomites in influenza transmission have long been discussed but remain unresolved [43], [44].

### Alternative explanations of human H5N1 patterns

Seasonality of human H5N1 was observed but not statistically significant in Egypt, while in Indonesia H5N1 incidence did not correlate with changes in meteorological variables. Therefore, it remains possible that the observed correlation between weather and human H5N1 in Egypt can be explained by chance. It is therefore important to evaluate possible alternative explanations and confounders. For instance, in Vietnam [45] and Thailand [46] H5N1 outbreaks in poultry have been linked to the occurrence of major festivals during which poultry is customarily consumed and transported between regions. However, events of this nature do not appear to have occurred repeatedly during periods of peak H5N1 incidence in humans in Indonesia or Egypt.

Alternatively, poultry rearing practices in these countries may differ seasonally; for example, backyard poultry farmers may move their animals indoors or to coops during rainy or cooler periods and allow poultry to range more freely during dry or more temperate periods. Indeed, a recent survey of backyard poultry owners in Egypt found that only 45.8% kept birds exclusively in cages outside the home [47] at any point in the year, and over 12% of owners kept poultry uncaged and inside the home at all times. By contrast, a second study across Indonesia found that 61–97% of poultry farmers never allowed poultry indoors [48]. In Tangerang, Indonesia, the district with the most H5N1 human cases, only 16% of farmers ever allowed poultry in the house [48]. Unfortunately, although these studies were quite comprehensive, neither asked about seasonal changes in practices and such changes cannot be ruled out as an explanation for observed trends. In addition, no information is available on the poultry handling practices of infected individuals or their close family.

Even if poultry handling practices do not change seasonally, such practices may interact with meteorological factors to increase the risk of human infection. For example, when asked in 2007 how they typically disposed of dead poultry prior to the outbreak of avian influenza, 48.2% of Egyptian backyard poultry owners reported that they threw the birds in the street and 16.8% threw dead birds in the canal [47]. In addition, 38% of Egyptian backyard poultry owners reported that they would slaughter and cook the remaining birds if some of their flock died from avian influenza symptoms [47]. Of owners in this group, 12.5% would throw wastes from the slaughter into the street, while 22.5% would throw the wastes into the canal [47]. It is possible that dead birds or offal in the street or canals may lead to increased risk of human H5N1 infection under specific weather conditions, that carcass disposal practices may vary between dry and rainy seasons, or that consumption of poultry, and thus butchering or handling of raw poultry mean, increases seasonally.

In Egypt, few if any human cases have occurred in large agricultural settings; human H5N1 cases were more commonly associated with small farm or backyard poultry outbreaks [49]. Incidence reports from Indonesia, on the other hand, suggest that large poultry operations have been associated with human H5N1 cases [49]. Visual inspection of commercial poultry outbreaks reported to the OIE by Egypt and Indonesia and human H5N1 time series appears to suggest that large scale poultry production is not a primary risk factor for human H5N1. However, since commercial poultry outbreaks occurred throughout the year in both Egypt and Indonesia and since under-reporting of H5N1 in poultry is likely high, the association between incidence patterns of H5N1 in commercial poultry and in humans cannot be clearly assessed from these data.

These analyses are necessarily limited by the quality of the H5N1 incidence data. These data are collected using largely un-evaluated surveillance systems and vary in completeness between countries, with some countries reporting cases in aggregate or omitting information such as age, date of onset, or location from case reports. However, at least for human H5N1, case reports from Indonesia and Egypt appeared generally complete up to May 2008; after this date, Indonesia declared the intention to report cases bi-annually and in aggregate [50]. In our analyses, we attempted to minimize the effect of data limitations: Ten-day intervals were used in order to minimize the effect of variation in reporting dates of symptom onset or hospitalization; and human incidence data was coded as a binary variable in order to minimize error due to potential under- or over-reporting of cases clustered in time.

In addition, the limited number of cases made it necessary to aggregate data across each country. The use of country-wide averages for temperature, precipitation, absolute humidity and relative humidity may have resulted in the use of conditions that do not reflect the actual conditions where a given case occurred. However, the impact of this limitation is likely greatest in Indonesia, where weather, especially temperature, varied more widely across the country during the study period. In Egypt, there was less variation between weather stations on environmental variables, and analysis of human incidence and weather in the Nile Delta region only resulted in similar findings.

### Conclusions

H5N1 incidence in humans oscillated seasonally in Egypt in 2006 to 2008 with a period of 14 months, and in Indonesia in 2005–2008 with a period of 18 months. By contrast, H5N1 incidence in commercial poultry was estimated to oscillate in Egypt with a period of 7 months (2006–2008) and did not show significant oscillation in Indonesia between 2005 and 2006. Peak human H5N1 incidence in Egypt appears to occur two months later each year, while in Indonesia incidence may peak every other year, with each peak occurring six months later in the year than the previous peak. Periods of highest risk for H5N1 infection could potentially be predicted from these data; however, validation of this model with more recent incidence data is needed to verify these results.

The calculated seasonality of human H5N1 incidence is consistent with existing reports that seasonal influenza (H1N1 and H3N2) incidence in humans varies in number and timing of peaks, and suggests that routine surveillance for influenza in tropical and sub-tropical regions should not be limited to winter months, as it is in temperate climates. Dushoff *et al.* [21] suggested that seasonal changes in transmission rate of seasonal influenza in humans are below measurement error and may be amplified by resonance between the natural period of intrinsic oscillation (determined by transmission parameters) and the seasonality. As a result, it has been suggested that a clear understanding of the contribution of weather to influenza seasonality may be impossible from modeling seasonal influenza due to the challenge of assessing actual morbidity levels. Our results suggest that analysis of novel influenza strains, such as H5N1, may bypass these difficulties and allow direct assessment of influenza seasonality and associated drivers.

In Egypt, human H5N1 peak incidence was preceded by and coincided with low precipitation and absolute humidity, and moderate temperature and relative humidity. In Indonesia, there was no clear relationship between weather and H5N1 incidence in humans. The association between human H5N1 incidence and low precipitation in Egypt supports previous findings that poultry H5N1 is associated with reduced annual rainfall [11], but seems to contradict findings that poultry H5N1 is associated with areas with high standing water or proximity to streams and rivers [13], [14], [17]. However, none of these previous studies assessed seasonal changes in water volume and cannot be directly compared to our findings. Further research using fine-grained meteorological and incidence data may help resolve some of these issues.

Our findings confirm laboratory and other published results that absolute humidity is a primary driver of influenza transmission seasonality, and suggest that transmission of H5N1 via fine particle aerosols is unlikely to have been the primary mode of human infection with H5N1 in Egypt; instead, meteorological conditions appear to have favored droplet transmission, such as may occur in during close contact between humans and infected poultry. This suggests that personal protective equipment, such as face masks, and protective behaviors, such as hand washing, commonly used for seasonal influenza prevention may also be useful for reducing the risk of infection with H5N1 for those in contact with potentially infected animals, including at live-bird markets.

## Methods

### Datasets

Human case data were obtained from the World Health Organization (WHO) Epidemic and Pandemic Alert and Response website [49] beginning in July 2005 for Indonesia and in March 2006 for Egypt, and continuing until May 1^st^ 2008. As of May 1, 2008, the majority of human H5N1 cases had occurred in Indonesia (n = 133), Vietnam (n = 106) and Egypt (n = 50). However, detailed information on cases in Vietnam was more limited than in Indonesia or Egypt, and cases occurring in Vietnam were therefore excluded from these analyses. Data on age, sex, date of onset, date of hospitalization, date of death (if fatal), country and region, and suspected exposure source were extracted from all case reports of human H5N1 in Indonesia and Egypt through May 1^st^, 2008. Date of symptom onset or hospitalization was available for 92% of cases in both Indonesia (n = 122) and Egypt (n = 46); all reports which contained information on date of symptom onset or hospitalization were included in the final dataset.

For analysis, cases were grouped by country into ten-day intervals beginning on Jan 1^st^ 2005 in Indonesia or Jan 1^st^ 2006 in Egypt, until May 1^st^, 2008 using date of symptom onset, or date of hospitalization when symptom onset was not available; a total of 122 intervals in Indonesia and 86 in Egypt. Ten-day intervals were used to limit the effect of combining symptom onset and hospitalization dates, as well as to account for the presumed incubation period of 3–10 days for influenza H5N1 [51] and the average duration of fatal illness of approximately 10 days – the average length of illness for 144 human H5N1 fatalities worldwide with date of onset and death available was calculated as 9.88 days. Due to the small sample size of H5N1 cases in each country, and to limit the impact of potential over- or under-reporting of clustered cases, incidence was recoded as a binary variable for Fourier analysis, indicating the occurrence of 0 or 1+ cases during each ten-day interval.

Weather data were obtained from the US National Oceanic and Atmospheric Administration's National Climatic Data Center (NCDC) web-based database [52]. Initially, data from all weather stations in each province (Indonesia) or governorate (Egypt) where at least one case occurred were downloaded from Jan 1st, 2005 to May 1st, 2008. For analysis, only stations with data available for the entire period were used. Weather data, collected daily by the NCDC, were averaged between all stations within an analysis region on a daily basis and then averaged across each ten-day interval. The variables extracted from weather station datasets were mean temperature, mean dewpoint and mean precipitation. In addition, relative humidity was calculated following Lawrence, 2005 [53], and vapor pressure was calculated, as a measure of absolute humidity, using the Clausius-Clapeyron equation [54], following Shaman & Kohn [37].

### Analysis

Although Poisson regression is typically used to analyze trends in influenza and other infectious diseases [55], [56], this method requires several assumptions which the H5N1 and meteorological datasets violate, including log-linearity and homoskedasticity [57]. In addition, the meteorological variables were highly multicollinear, which can limit the utility of Poisson regression [58]. Overall, Poisson regression was determined to be inappropriate for this dataset; instead, analysis of incidence seasonality was performed using Fourier analysis time series methods [59], [60].

For analysis, periodograms were smoothed using Tukey-Hanning weights [61]. For each country, Fourier analysis was performed for each variable individually, and multiple Fourier analyses (cross-spectra) were used to compare the periodicity of human cases with the other variables. Statistical significance was computed using Bartlett's Kolmolgorov-Smirnov test to evaluate the significance of the entire periodogram relative to white noise [62], [63], and Fisher's kappa test for periodicity to evaluate the significance of the maximum periodogram ordinate [60], [62], [63]. Fisher's kappa test is a standard test used to determine periodicity in time series data and is based on the dominant frequency in the Fourier transformed data [64]. This test is sufficient for determining periodicity in data for which a single frequency of oscillation is hypothesized [60], [63], [64].

Sinusoidal equations describing each series were fitted to the data using the main frequency from individual Fourier analyses (Supplementary Table S1) and were then used for graphical comparisons of time series. Note that the equations for relative humidity, precipitation and human H5N1 incidence for Egypt, as well as those for temperature, precipitation, human H5N1 incidence and relative humidity for Indonesia, were scaled for graphing in order to allow visual comparisons on the same ordinate (y) axis. These equations (without scaling) were also used for calculating values of meteorological variables at peak H5N1 incidence and at 6–8 weeks prior to peak H5N1 incidence in humans, by first calculating the time (in units of ten-day intervals) of the first H5N1 incidence peak, and then by evaluating the sinusoidal equations for each of the meteorological variables at this value of x (abscissa).

Fourier analyses were performed using SAS (SAS Institute, Cary, NC) and graphs were produced using Mathematica 6 (Wolfram Research, Urbana -Champaign, IL). Calculations of squared coherencies and parameters for sinusoidal equations were performed using Mathematica 6 [60].

## Supporting Information

Figure S1 Fourier analysis periodogram for human H5N1 incidence in Egypt – periodogram ordinate versus frequency.
(TIF)

Figure S2 Fourier analysis periodogram for human H5N1 incidence in Indonesia – periodogram ordinate versus frequency.
(TIF)

Figure S3 Squared coherency (κ^2^) values versus frequency (x-axis; cycles per year) for cross-spectra between human H5N1 cases and meteorological variables in Indonesia. (A) Precipitation; (B) Temperature; (C) Relative humidity; and (D) Absolute humidity.
(TIF)

Table S1 Equations for sinusoidal curves of time series data, using frequencies associated with the maximum periodogram ordinates for each data series. Equations were parameterized using based on a best fit with the data, using the Fourier analysis frequencies as a starting point for optimization.
(DOC)
