# Supplementary figures and images for: Seasonal Oscillation of Human Infection with Influenza A/H5N1 in Egypt and Indonesia
Source: PLoS One. 2011 Sep 1;6(9):e24042. doi: 10.1371/journal.pone.0024042 (PMC3164700; doi:10.1371/journal.pone.0024042)

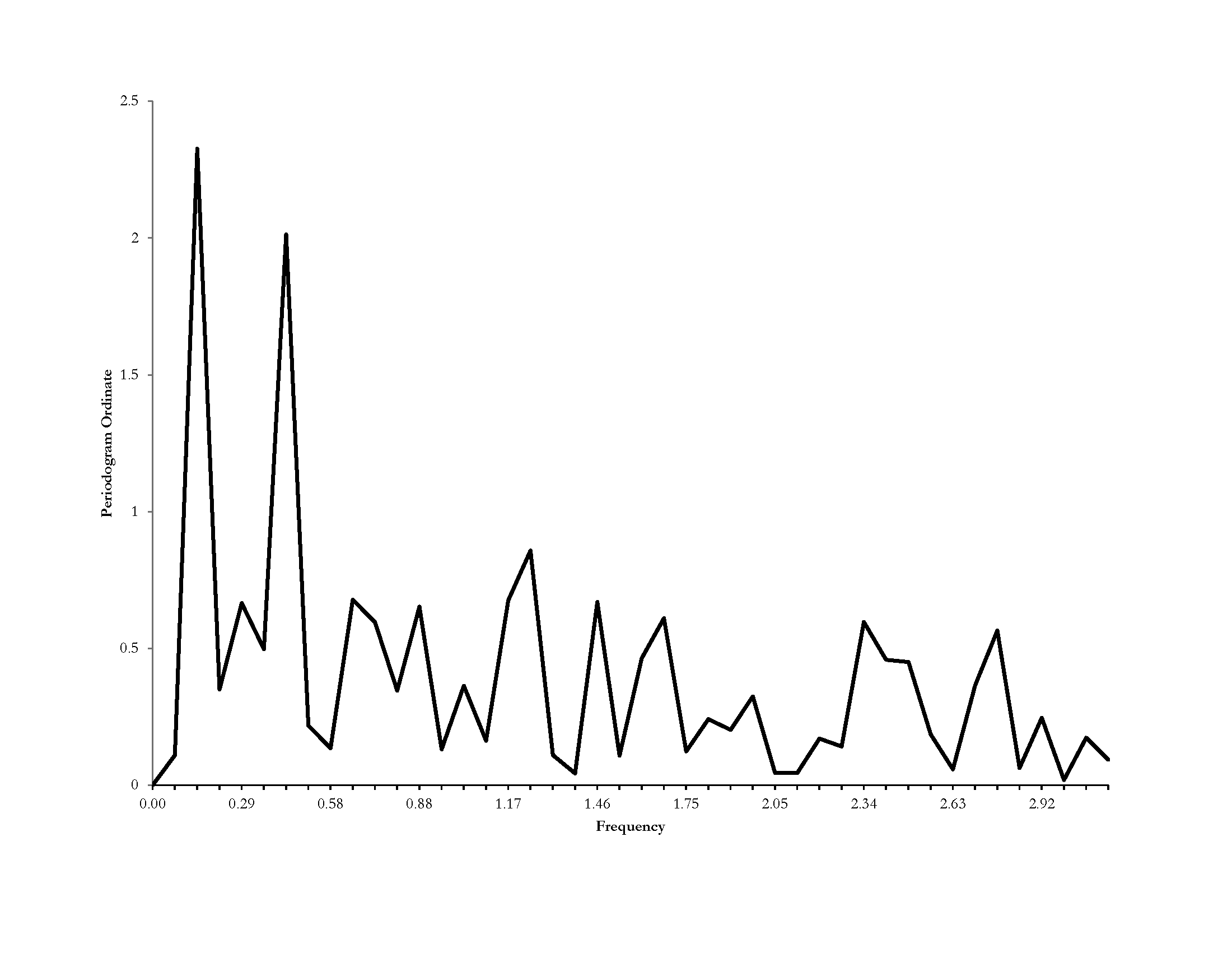

Supplement: Figure S1 — Fourier analysis periodogram for human H5N1 incidence in Egypt – periodogram ordinate versus frequency. (TIF) [file pone.0024042.s001.tif]

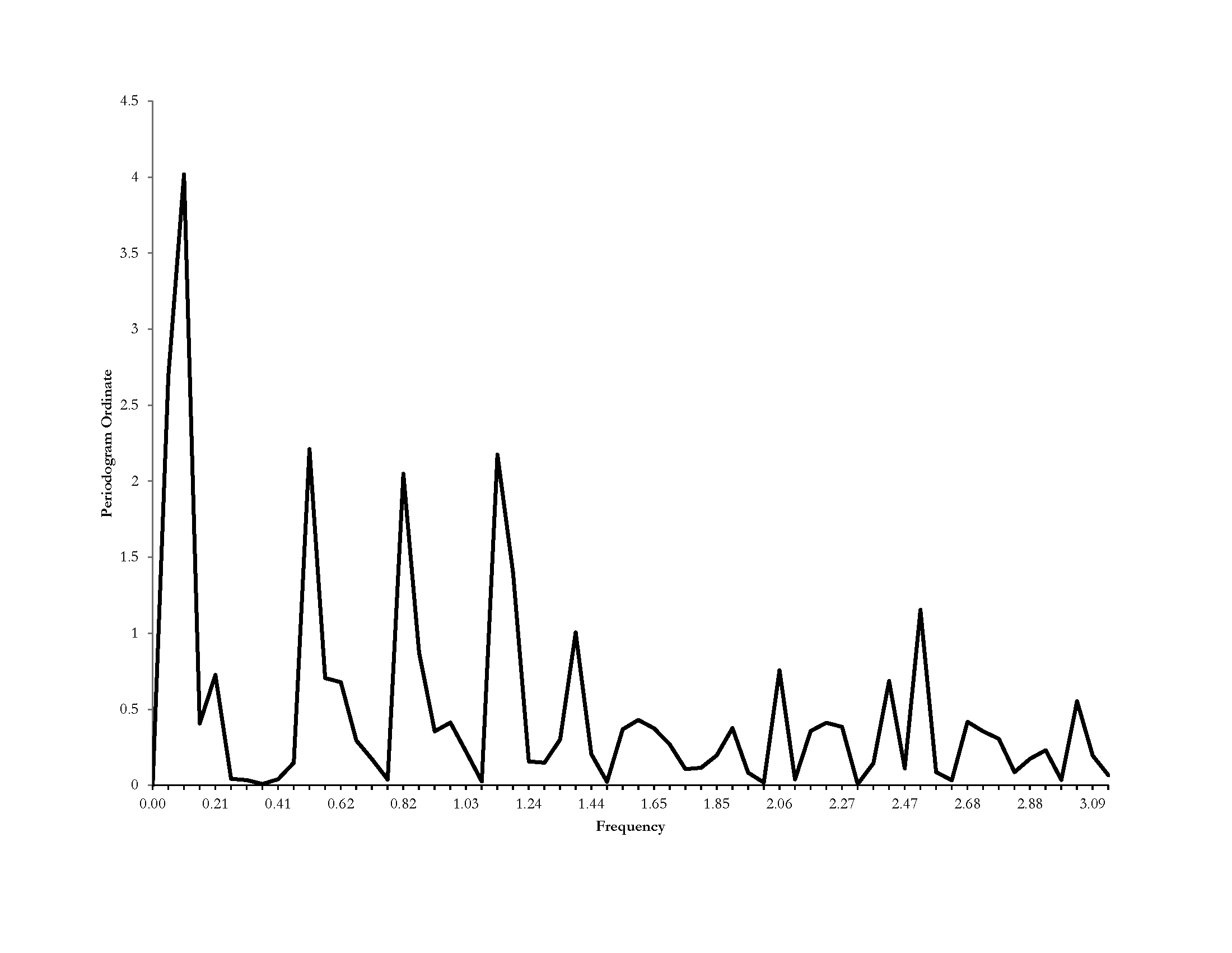

Supplement: Figure S2 — Fourier analysis periodogram for human H5N1 incidence in Indonesia – periodogram ordinate versus frequency. (TIF) [file pone.0024042.s002.tif]

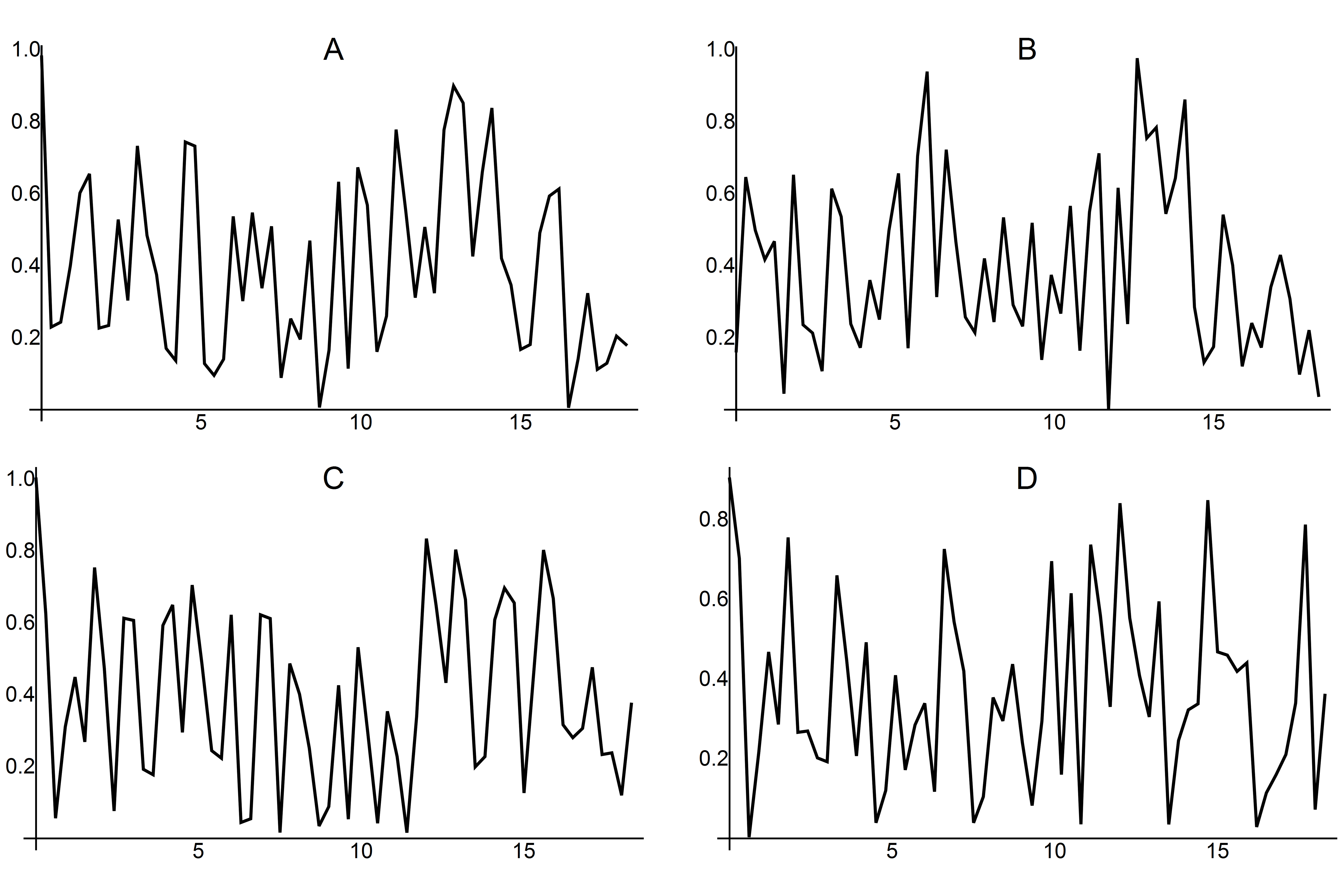

Supplement: Figure S3 — Squared coherency (κ2) values versus frequency (x-axis; cycles per year) for cross-spectra between human H5N1 cases and meteorological variables in Indonesia. (A) Precipitation; (B) Temperature; (C) Relative humidity; and (D) Absolute humidity. (TIF) [file pone.0024042.s003.tif]
