# Supplementary material for: Seasonal Oscillation of Human Infection with Influenza A/H5N1 in Egypt and Indonesia
Source: PLoS One. 2011 Sep 1;6(9):e24042. doi: 10.1371/journal.pone.0024042 (PMC3164700; doi:10.1371/journal.pone.0024042)
Supplement: Table S1 — Equations for sinusoidal curves of time series data, using frequencies associated with the maximum periodogram ordinates for each data series. Equations were parameterized using based on a best fit with the data, using the Fourier analysis frequencies as a starting point for optimization. (DOC) [file pone.0024042.s004.doc]

**Supplementary Tables:**

Table S1

| **Country** | **Data series** | **Best Fit Sinusoidal Equation** |
| --- | --- | --- |
| Indonesia | H5N1 Incidence | Incidence = 0.540062 + 0.266721 Sin[3.59297 + 0.110992 t] |
|  | Precipitation | Precipitation = 5.00292 + 2.9153 Sin[2.21148 + 0.154526 t] |
|  | Temperature | Temperature = 26.9218 + 0.391679 Sin[2.41693 + 0.360885 t] |
|  | Relative humidity | Relative Humidity = 78.203 + 4.16244 Sin[1.63332 + 0.154526 t] |
|  | Absolute humidity | Absolute Humidity = 28.057 + 1.09753 Sin[1.40235 + 0.155 t] |
| Egypt | H5N1 Incidence | Incidence = 0.250553 - 0.254814 Sin[0.0663672 - 0.172513 t] |
|  | Precipitation | Precipitation = 0.11132 + 0.174293 Sin[1.01284 + 0.176894 t] |
|  | Temperature | Temperature = 23.0225 + 8.23477 Sin[4.24837 + 0.172572 t] |
|  | Relative humidity | Relative Humidity = 50.9358 + 7.19296 Sin[1.80461 + 0.172947 t] |
|  | Absolute humidity | Absolute Humidity =14.9062 + 6.06039 Sin[3.98757 + 0.172893 t] |
